# Adverse health and environmental outcomes of cycling in heavily polluted urban environments

**DOI:** 10.1038/s41598-021-03111-3

**Published:** 2022-01-07

**Authors:** Ewa Adamiec, Elżbieta Jarosz-Krzemińska, Aleksandra Bilkiewicz-Kubarek

**Affiliations:** AGH University of Science and Technology, 30 Mickiewicza Av., 30-059 Kraków, Poland

**Keywords:** Environmental impact, Geochemistry

## Abstract

Cycling is a healthy habit; however, are its benefits outweighing risks when biking in heavily trafficked and air-polluted cities? Research involved studying contamination with traffic-related elements of dust collected from bike paths located in top trafficked cities of Europe in Poland. Human health risk was assessed via inhalation and ingestion pathways for adults and children. Bike path dust was heavily contaminated with Zn, Cd (Geoaccumulation index Igeo 4) and Pb (Igeo 3), sourced predominantly from nonexhaust car emissions. The concentrations of metals in dust decreased in the following descending order: Zn > Mn > Cu > Pb > Cr > Cd. A fractionation study revealed that Zn and Cd are almost entirely bioavailable (Mobility factor MF above 90%), causing hazards to humans and the environment. The highest congested intersections result in more TRAP-contaminated dust deposited on bike paths, which is easily resuspended, posing a health risk for cyclists or pedestrians. Avoiding cycling in proximity to heavily trafficked routes should be considered, when possible, as well as physical removal of dust by wet sweeping to limit dust resuspension.

## Introduction

There is a well-established worldwide trend of investing in the development of infrastructure for cyclists, which brings many obvious and positive outcomes for cities’ residents in terms of increasing their quality of life or simply by delivering tangible benefits to the environment. The link between physical activity in urban environments (such as cycling or jogging) and enhanced exposure to air pollutants was previously confirmed by multiple authors^1,2^. It was found that while physical activity brings many health benefits, when performed outdoors in polluted environments, it can increase exposure to air pollutants^3–5^, causing multiple possible health outcomes. De Hartog et al.^6^, for instance, reported that cycling in urban areas contributes even stronger to health and safety risks owing to potentially high levels of traffic density and exposure to air pollution and road traffic noise. The results of Apparicio et al.^7^ revealed, for instance, that cyclists are exposed to 47 µg/m^3^ NO_2_ and 3.3 dB(A) more when cycling on a primary road than on a residential street. This abovementioned research is especially important since it was conducted in one of the most polluted cities in the world, Delhi India. De Hartog et al.^6^ concluded that air pollution in Delhi should be considered a major health concern for cyclists. Other research conducted on cyclists’ exposure to air pollution in urban environments was mostly conducted in cities with much better air quality than in India or Poland. Based on the 2019 study^8^, Poland is one of the most polluted countries in Europe in terms of air quality. Considering 100 cities with the worst air quality in Europe, as many as 29 are in Poland^8^. At the same time, cycling grid infrastructure is constantly growing in Poland, especially in urban environments, when bike paths are commonly in proximity to the main routes.

Richardson et al.^9^ noticed a correlation between low incomes, bad air quality and mortality in East Europe when compared to Western European countries. The same correlation can be observed in Poland, where the prevailing number of old cars driving in city traffic directly relates to enhanced traffic-related air pollution (TRAP).

In general, TRAP is considered a major source of pollution in any urban environment, especially in proximity to heavily congested roads. However, it must also be stated that this term refers not only to air pollution resulting from the combustion of car fuel but also to nonexhaust sources of pollution, which results from the abrasion of tires, clutches or brakes or even road surface infrastructure^10–16^. In fact, according to some researchers^17,18^, nonexhaust emissions related to transport prevail in the urban environment over exhaust emissions and can be accountable for as much as 90% of all traffic-related air pollution.

Road dust is the main constituent of bike path sediment or sidewalk dust and comprises both geogenic and anthropogenic particles, which are deposited in proximity to roads. As indicated by many authors^13,19^, road (street) dust is severely enriched with heavy metals, especially Cd, Cr, Cu, Ni, Pb and Zn. Many authors^20–25^ predict that approximately 70% of brake-emitted metals undergo secondary resuspension into the atmosphere.

Nonexhaust emissions from cars have been proven by many authors to be linked to very serious health issues for residents of the cites. Our previous research^13^ was conducted to assess the human health risk associated with the exposure of pedestrians and city residents, such as children and adults, to the finest fraction of dust collected in proximity to trafficked roads. Our findings were in line with the outcomes of other authors^26,27^ who concluded that children are at much greater health risk when inhaling dust since the dose is twice as high as that taken by adults since children breath approximately 50% more air per kg of their body weight than adults, and their respiratory system is not fully formed.

Despite the fact that road dust is well recognized as a major source of noncombustion air pollution, most research is still focused mainly on its chemical composition rather than on the mobility of heavy metals entrapped in the dust. Information regarding the total concentration of metals in the dust deposited in bike paths is negligible and thus insufficient to draw appropriate conclusions on its potential health implications for city residences and the toxicity of the dust to the environment. The environmental and health effects of contaminants in dust are inextricably linked to its mobility. It is therefore especially important to determine speciation studies regarding traffic-related metals in bike path dust, which is situated near main roads in cities, to better understand the health risks posed by airborne dust for humans and the environment.

Thus, the mobility and bioavailability of contaminates are governed and controlled by various physicochemical parameters, e.g., pH, redox potential Eh, temperature, organic matter content or content of oxides acting as sorbents^28–30^. Heavy metals that are bound in soluble and exchangeable fractions are easily mobile and thus most bioavailable; hence, they pose the highest risk to biota^31^ and to all components of the environment, that is surface water, groundwater or soil^32–34^. The most suitable method to determine and identify forms of metals and their binding strength and reactivity in various environmental samples are sequential extraction methods. Different methodologies are employed and commonly used for that purpose, mostly BCR (Community Bureau of Reference), Tessier or VI step sequential extraction protocols. There are multiple studies on the chemical or mineralogical characteristics of road dust, while there is substantially less research conducted on sidewalk and bicycle path sediment or even fewer fractionation studies. Considering that cyclists are especially susceptible to road and bike path dust exposure (via all pathways), it is especially important to perform a detailed fractionation study. Owing to the toxicity of dust deposited in proximity to heavily congested cities’ main roads due to intensified start/stop activities, it should be predicted that cycling or jogging in such polluted areas may cause a health threat. This is the main objective of the study to determine geochemical composition and thus contamination with heavy metals of dust collected from bike trails situated in proximity to heavily congested roads in the three largest and one of the most air polluted and congested cities of Poland and consequently in Europe. To estimate the bioavailability of toxic elements in bike paths, a dust VI step fractionation study was conducted followed by mobility factor MF calculation^35–39^. All analyses were conducted only on the finest fraction of bike trail dust < 20 µm since our previous findings^40^ revealed that 87.38% of the emitted particulates from brake pads are of diameters under 20 µm; moreover, this fraction is considered representative in terms of its high mobility and bioavailability to both humans and the environment. Furthermore, a human health risk assessment was calculated to determine the risk associated with the exposure of cyclists (both adults and children) to airborne dust.

## Materials and methods

### Sampling area

Research was conducted in four Polish cities: Warsaw Capital of Poland, the largest and most populated city; Krakow, the second largest and most populated city; Wrocław, the 4th most populated city; and Opole, the medium sized city (ranked 27th largest city of Poland. The same variables were considered when selecting research sites, i.e., extensive network of bike paths situated specifically in proximity to heavily congested roads, high traffic intensity, intersection areas where there is enhanced start-stop and braking activities as well as poor ambient air quality. Research sites were chosen carefully, as these cities were ranked as the most congested cities of Poland and Europe and characterized by rapid development of bicycle infrastructure. Based on the annual Traffic Index report provided by TomTom for 2020 (year of sampling campaign), out of 166 studied European cities, Kraków was the second most congested city in Europe in the category of cities below 800,000 residents according to the annual Traffic Index report prepared by TomTom, with a congestion level of 36%, and Wrocław was the 3rd most congested city of Europe, with a 35% congestion level in 2020. Warsaw in the category of large cities above 800 thousand residents was ranked as the 17th most congested city of Europe, with a congestion level of 31%. The Opole, as much less populated and trafficked city of Poland (with only 128 012 inhabitants in 2020), was not included in the TomTom ranking. However, this city’s sampling sites were considered a reference point in this study.

**Figure 1:**
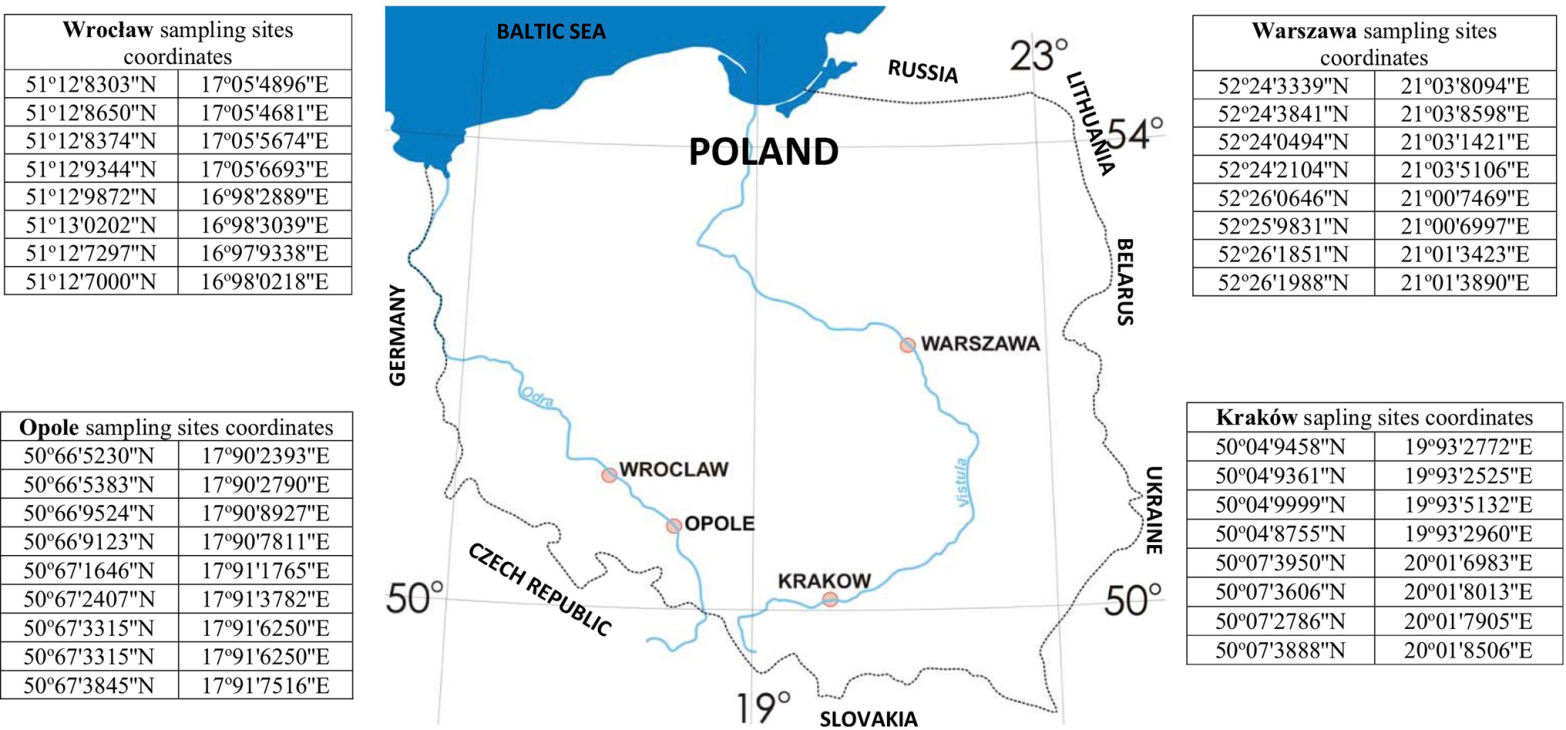
Sampling sites location and geographical coordinates.

Total of 32 samples of dust were collected. Detailed sampling site coordinates are depicted in Fig. 1.

Dust was collected from every sample location from 4 sides of the intersection, and each section of the bike path 20 m long was then swept using a brush. Approximately 0.5 kg of dust from every site location was averaged and homogenized for further analysis. All samples were collected over a period of one week to maintain similar weather conditions. There was no precipitation during sampling, there was a weak wind from the west (wind speed ranged from 2 to 5 km/h) and the ambient temperature was between 12 and 16 °C, air humidity 71%.

## Methodology

### Total concentration of contaminants in the dust

For the purpose of this study, only heavy metals considered tracers of traffic-related air pollution emissions (that is, Cu, Pb, Zn, Cr, Mn and Cd) were analyzed in the fraction below 20 µm of bike lane dust samples. As previously stated, the finest fraction of dust was selected since our prior findings^40^ revealed that 87.38% of the emitted particulates from brake pads are of diameters under 20 µm, and this fraction is considered representative in terms of its high mobility and bioavailability to both humans and the environment. Dust was digested in concentrated nitric acid using a microwave according to U.S. EPA Method 3050B^41^ and further chemically evaluated via the ICP-MS method (ELAN 6100; Perkin Elmer according to U.S. EPA Method 6020B^42^) at the accredited analytical laboratory at the Faculty of Geology, Geophysics and Environmental Protection, AGH University of Science and Technology. Appropriate quality control of the analysis was then employed by using reagent blanks and certified reference materials BCR 723 (road dust), ERM-CZ120 (fine dust PM10-like, EU JRC IRMM), METRANAL^TM^32 (light sandy soil, grain size 100 µm Analytika Czech Republic) and SRM 1848a (urban particulate matter), including a fractionation study protocol. The analytical bias was found to be statistically insignificant (p = 0.05).

### Geoaccumulation index, Igeo

The total concentrations of metals in the finest fraction (< 20 µm) of bike path dust were then compared with the geochemical background based on Turekian and Wedephol^43^, employing as a reference background value—World average shale value (ASV) for Cd (0.3 mg/kg), Cu (45 mg/kg), Cr (90 mg/kg), Pb (20 mg/kg), Mn (850 mg/kg) and Zn (95 mg/kg).

The geo index was calculated according to the equation provided by Müller^44^:

$${\text{I}}_{{{\text{geo}}}} = {\text{log}}_{{2}} \cdot \left( {{\text{C}}_{{\text{n}}} /{1}.{5} \cdot {\text{B}}_{{\text{n}}} } \right)$$

where Cn is the concentration of element n and Bn is the geochemical background value.

### Chemical fractionation study

A fractionation study was conducted on fractions below < 20 µm of bike lane dust, since the finest fraction is regarded as especially dangerous for human health and the environment. A chemical fractionation study was conducted using a modified VI step sequential extraction protocol according to Salomons and Fӧrstner^45^; however, instead of HNO_3_, *aqua regia* was used as an extraction solution in the VI step. One gram of air-dried sample was subjected to leaching according to the fractionation protocol presented in Table 1.

**Table 1:** Chemical fractionation protocol.

Step	Fraction	Targets phases	VI steps protocol
1	Exchangeable cations	Soluble, cation exchangeable forms of elements	1 M ammonium acetate, pH 7 solid/solution ratio 1:20, 2 h shaking time
2	Carbonates	Carbonates	1 M sodium acetate buffer, pH 5 (Tessier et al. 1979); solid/solution ratio 1:20, 5 h shaking time at 20 °C
3	Easily reducible phases	Mn oxide, partly amorphous Fe-oxyhydrates	0.1 M NH_2_OH*HCl + 0,001 M HNO_3_, pH 2 (Chao 1972), dilution 1:100, 12 h shaking
4	Moderately reducible phases	Amorphous and poorly crystallized Fe-oxyhydroxides	0.2 M ammonium oxalate + 0,2 M oxalic acid, pH 3 (Schwertmann 1964), dilution 1:100, 24 h shaking time, pH 2
5	Organic fraction including sulphides	Organic matter and sulphides	30% H_2_O_2_ + HNO_3_, pH 2, 85 °C, extracted with 1 M ammonim acetate, dilution 1;100, 24 h shaking
6	Residual	Remaining, nonsilicate bound metals, e.g., detrital silicates, crystalline Fe-oxides	*Aqua regia* digestion

The concentrations of Cu, Pb, Zn, Cr, Mn and Cd in the obtained leachates from each fractionation step were analyzed using the ICP-MS method.

### Mobility factor

The mobility factor (MF) was calculated according to the equation provided by various authors^35,37,38,46^.

$${\text{MF}} = \left[ {{{\left( {{\text{F1}} + {\text{F2}} + {\text{F3}} + {\text{F4}}} \right)} \mathord{\left/ {\vphantom {{\left( {{\text{F1}} + {\text{F2}} + {\text{F3}} + {\text{F4}}} \right)} {\left( {{\text{F1}} + {\text{F2}} + {\text{F3}} + {\text{F4}} + {\text{F5}} + {\text{F6}}} \right)}}} \right. \kern-\nulldelimiterspace} {\left( {{\text{F1}} + {\text{F2}} + {\text{F3}} + {\text{F4}} + {\text{F5}} + {\text{F6}}} \right)}}} \right] \times 100\%$$

where F1 stands for the concentration of elements in exchangeable positions, F2 represents elements bound with carbonates, F3 represents Mn oxide and partly amorphous Fe-oxyhydrates, F4 represents amorphous and poorly crystallized Fe oxyhydroxides, F5 represents metals bound with organic matter fractions and F6 represents metals in the remaining residual fraction.

### Human health risk assessment

Human Health Risk Assessment was calculated by referencing USEPA guidelines and parameters provided in Table 2, according to Eqs. (1–4)

1 $${\text{ADDinh}} = \left( {{\text{C}} \times {\text{InhR}} \times {\text{EF}} \times {\text{ED}}} \right)/\left( {{\text{PEW}} \times {\text{BW}} \times {\text{AT}}} \right)$$

2 $${\text{ADDingest}} = ({\text{C}} \times {\text{IngR}} \times {\text{EF}} \times {\text{ED}} \times {\text{CF}})/\left( {{\text{BW}} \times {\text{AT}}} \right)$$

3 $${\text{HQing}} = {\text{ADDingest}}/{\text{RfDing}}$$

4 $${\text{HQinh}} = {\text{ADDinhalation}}/\left( {{\text{RfDinh}} \times {1}000\;\upmu {\text{g}} \times {\text{mg}}^{{ - {1}}} } \right)$$

**Table 2:** Parameters used to assess human health risk^40,46–51^.

Parameters	Descriptions	Children	Adults
BW	Average body weight (kg)	20.3	70
EF	Exposure frequency (days)	1 h per 180 days (due to favorable weather conditions) which accounts for 7.5 days per year	1 h per 180 days (due to favorable weather conditions) which accounts for 7.5 days per year
ED	Exposure duration (years)	6	24
IngR	Ingestion rate for soil expressed in mg/day	100	50
CF	Conversion factor (kg/mg)	10^−6^	10^−6^
PEF	Partical emission factor (m^3^/kg)	1.316 × 109	1.316 × 109
InhR	Inhalation rate (10m^3^/day)	7.6	20
AT	Average time (ED × 365 days)	6 × 365	24 × 365

Potential adverse health effects may occur when the HQ index exceeds the threshold value of 1. The greater the value of HQ above unity, the greater the level of concern^26,52^. The hazard index (HI) was then calculated as a sum of individual HQs, indicating an overall potential noncarcinogenic effect posed by more than one deleterious substance. No significant risk is stated when HI < 1, but when HI > 1, chronic risk is more likely to occur.

## Results and discussion

### Contamination of bike path dust with traffic-related elements (TRAPs)

All dust collected from bike paths located in proximity to main roads in four main cities of Poland was highly enriched with heavy metals. The concentrations of metals decreased in the following descending order: Zn > Mn > Cu > Pb > Cr > Cd. Only in Opole, which is a significantly less trafficked city, did the composition of dust differ and was predominantly enriched with Mn, Zn, Pb, Cu, Cr and Cd. Dust collected from Kraków was 41% more contaminated with Cu and 101% and 102% more contaminated with Zn and Cd, respectively, than dust collected in Opole. Dust from Warsaw was 150% more contaminated with Cu and 170% more contaminated with Zn and Cd when compared to the concentration of metals in dust in Opole. Dust collected from bike paths in Wrocław was also significantly more contaminated with all heavy metals than dust in Opole, 47% more Cu, 167% more Zn and 14% more Cd. Detailed results of metal concentration in bike path dust samples are included in Table 3.

**Table 3:** Concentration of Cu, Pb, Zn, Cr, Mn and Cd in fraction < 20 µm of bike trail dust.

Localization Number of sample	Cu	Pb	Zn	Cr	Mn	Cd
	Mean concentration [mg/kg]					
Kraków (n = 8)	343.5	261.5	2355	158	1360	6.075
Warszawa (n = 8)	609.5	216	1890	215	1325	4.885
Wrocław (n = 8)	358	274.5	1870	183	1039.5	3.14
Opole (n = 8)	243.5	295.5	1120	120	1445	2.75

**Figure 2:**
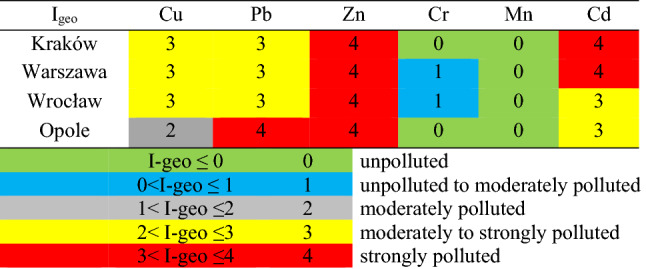
I_geo_ classification of dust.

When comparing the delivered results on individual metal concentrations with the background values for specific metals, it was found that dust collected from bike paths in Kraków, Warsaw and Wrocław was moderately to strongly polluted with Cu and Pb (I_geo_ 2–3) and strongly polluted with Zn and Cd. However, dust collected in all cities was classified as unpolluted with Cr and Mn (I_geo_ 0–1). Dust from Opole was moderately to strongly polluted with Pb, Zn and Cd whereas unpolluted with Cu. Cr and Mn. The results of the I_geo_ calculation are presented in Fig. 2.

### Mobility and bioavailability of contaminants in dust

The mobility of individual contaminants was determined based on a fractionation study (presented in detail in Fig. 2) and MF calculations. Metal bioavailability in dust from bike paths decreased in the following order: Cd > Mn > Zn > Pb > Cr > Cu. Copper, lead and chromium in dust were predominantly bound with residual fractions as well as organic matter and sulfates. Thus, the low bioavailability and mobility of this contaminant in the environment. Copper was bound with stable fractions in 79 to 90%. Pb from 66 to 89% and chromium from 71 to 88%. Only 12% of the total Cu was mobile in dust collected from Kraków, 10% in dust from Warsaw, 19% in dust from Wrocław and 21% of Cu was potentially labile in dust from Opole. Zinc, Mn and highly toxic Cd were much more labile since they are predominantly bound with easily exchangeable fractions, carbonates as well as Fe/Zn oxides. Only 5 to 17% of Zn in all dust samples is bound with organic matter and residuum. 5–12% of Mn as well as 2–10% of Cd.

**Figure 3:**
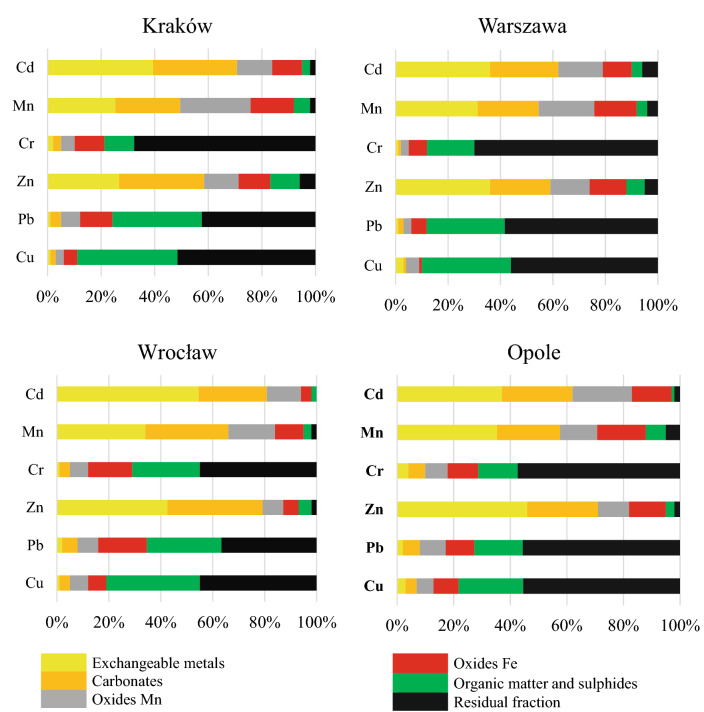
Results of dust fractionation study.

A fractionation study confirmed that Zn and Cd, which were found in extreme concentrations in bike path dust (Igeo indices above 3), are almost entirely bioavailable and mobile, thus causing direct hazards to humans and the environment. Detailed fractionation study results are presented on Fig. 3, whereas mobility factor calculation results are depicted in Table 4.

**Table 4:** Mobility factor (%) results.

MF	Cu (%)	Pb (%)	Zn (%)	Cr (%)	Mn (%)	Cd (%)
Kraków	12	25	83	21	91	95
Warszawa	10	12	88	12	92	90
Wrocław	19	34	94	30	95	98
Opole	21	27	95	28	87	97

### Human health risk assessment

The results of noncancerous human health risk calculations associated with the ingestion and inhalation contact of both adults and children with bike lane dust are depicted in Table 5.

**Table 5:** Noncancerogenic human health risk estimation for children and adults with bike path dust.

Elements		Cd	Cr	Cu	Mn	Pb	Zn	HI
**Children**								
Inhalation								
Kraków	ADD	4.99E-08	1.30E-06	2.82E-06	1.12E-05	2.15E-06	1.94E-05	
	HQ	4.99E-13	3.71E-11	1.13E-07	5.58E-10	7.56E-09	5.80E-06	5.92E-06
Warszawa	ADD	4.01E-08	1.76E-06	5.01E-06	1.09E-05	1.78E-06	1.55E-05	
	HQ	4.01E-13	5.05E-11	2.01E-07	5.44E-10	6.24E-09	4.65E-06	4.87E-06
Wrocław	ADD	2.58E-08	1.50E-06	2.94E-06	8.53E-06	2.26E-06	1.54E-05	
	HQ	2.58E-13	4.30E-11	1.18E-07	4.27E-10	7.93E-09	4.61E-06	4.73E-06
Opole	ADD	2.26E-08	9.85E-07	2.00E-06	1.19E-05	2.43E-06	9.21E-06	
	HQ	2.26E-13	2.82E-11	8.05E-08	5.93E-10	8.56E-09	2.76E-06	2.85E-06
Ingestion								
Kraków	ADD	3.94E-06	1.02E-04	2.23E-04	8.81E-04	1.69E-04	1.53E-03	
	HQ	2.16E-09	1.72E-07	5.25E-06	1.18E-05	2.81E-07	1.96E-05	3.70E-05
Warszawa	ADD	3.17E-06	1.40E-04	3.95E-04	8.59E-04	1.40E-04	1.23E-03	
	HQ	3.17E-09	4.18E-07	1.58E-05	2.06E-05	4.90E-07	3.68E-05	7.41E-05
Wrocław	ADD	2.04E-06	1.19E-04	2.32E-04	6.74E-04	1.78E-04	1.21E-03	
	HQ	2.04E-09	3.56E-07	9.29E-06	1.62E-05	6.22E-07	3.63E-05	6.28E-05
Opole	ADD	1.78E-06	7.78E-05	1.58E-04	9.36E-04	1.92E-04	7.26E-04	
	HQ	1.78E-09	2.33E-07	6.31E-06	2.25E-05	6.70E-07	2.18E-05	5.15E-05
**Adults**								
Inhalation								
Kraków	ADD	3.57E-08	9.26E-07	2.02E-06	7.98E-06	1.54E-06	1.39E-05	
	HQ	3.57E-13	2.66E-11	8.11E-08	3.99E-10	5.40E-09	4.15E-06	4.24E-06
Warszawa	ADD	2.87E-08	1.26E-06	3.58E-06	7.78E-06	1.27E-06	1.11E-05	
	HQ	2.87E-13	3.61E-11	1.44E-07	3.89E-10	4.47E-09	3.33E-06	3.48E-06
Wrocław	ADD	1.85E-08	1.07E-06	2.10E-06	6.10E-06	1.62E-06	1.10E-05	
	HQ	1.85E-13	3.07E-11	8.45E-08	3.06E-10	5.68E-09	3.30E-06	3.39E-06
Opole	ADD	1.62E-08	7.05E-07	1.43E-06	8.49E-06	1.74E-06	6.58E-06	
	HQ	1.62E-13	2.02E-11	5.75E-08	4.24E-10	6.11E-09	1.98E-06	2.04E-06
Ingestion								
Kraków	ADD	2.14E-06	5.57E-05	1.21E-04	4.79E-04	9.22E-05	8.30E-04	
	HQ	2.14E-09	1.67E-07	4.84E-06	1.15E-05	3.23E-07	2.49E-05	4.17E-05
Warszawa	ADD	1.72E-06	7.58E-05	2.15E-04	4.67E-04	7.61E-05	6.66E-04	
	HQ	1.72E-09	2.27E-07	8.59E-06	1.12E-05	2.67E-07	2.00E-05	4.03E-05
Wrocław	ADD	1.11E-06	6.45E-05	1.26E-04	3.66E-04	9.67E-05	6.59E-04	
	HQ	1.11E-09	1.94E-07	5.04E-06	8.79E-06	3.39E-07	1.98E-05	3.42E-05
Opole	ADD	9.71E-07	4.23E-05	8.59E-05	5.09E-04	1.04E-04	3.95E-04	
	HQ	9.71E-10	1.27E-07	3.43E-06	1.22E-05	3.65E-07	1.19E-05	2.80E-05

It was found that the highest noncarcinogenic risk associated with the exposure of children and adults to TRAP elements in bike lane dust is via the ingestion pathway. HQ_ing_ was the highest with respect to Cu, Mn and Zn, thus indicating that ingestion of dust rich with these metals will cause the highest health threat to both children and adults. Inhalation of dust particles was then the second most health-hazard route of chemical exposure. The highest HQ_inh values_ for both children and adults were found with respect to Zn, Cu and Pb. The highest noncancerous human health risk was associated with the exposure of cyclists to dust in Kraków (the second most trafficked city in Europe), where the risk was one order of magnitude higher than that in Warsaw and Wrocław and significantly higher than that in the least trafficked city of Opole. The results revealed that children are approximately 50% more susceptible to risk due to dust exposure than adults. In conclusion, however, the limit value for HQ = 1 was not exceeded for noncarcinogenic risk in any of the cities studied.

We must be aware that due to the limited availability of data and indicators provided by the EPA and the literature sources, we were able to assess only partial health risks associated with selected TRAP elements in dust to which cyclists are potentially exposed when biking in urban environments. The EPA does not formulate any guidelines or factors specifically for assessing or health risk for active people in motion during physical activity. Such an important derivative, e.g., cyclist riding speed, was not considered even though, for instanceBigazzi et al.^53^ indicate that when exceeding the optimal cyclist riding speed of 12 and 20 km per hour on city roads, by more than 10 km/h, this will result in a double inhalation dose over a fixed distance.

## Conclusions

Dust collected from bicycle paths in heavily trafficked cities of Poland was strongly contaminated with TRAP metals, especially Zn and Cd. Health risk related to dust exposure for cyclists via exposure pathways (inhalation and ingestion contact) is directly proportional to the congestion level in individual cities. The highest congested intersections result in more TRAP-contaminated dust deposited on bike paths, which is easily airborne and undergoes resuspension, thus causing an enhanced health risk for cyclists. A fractionation study confirmed that Zn and Cd, which were found in extreme concentrations in bike path dust (Igeo values above 3), are almost entirely bioavailable and mobile, thus causing direct hazards to humans and the environment. Considering that there are also multiple other Cd and Pb sources and exposure pathways for humans, the obtained results should be regarded as alarming. As previously stated it should also be noted that the health risk for cyclists in the urban environment assessed in this study is underestimated due to the limited availability of data and indicators provided by the EPA.

Moreover, our research was conducted during the COVID pandemic, which itself has caused a significant drop in urban congestion levels around the world. As stated in the TOMTOM report, some cities experienced as many as 30 days with low traffic—where congestion levels were at least 50% lower than the same day in 2019. The TomTom report revealed that in Polish cities such as Warsaw, Kraków and Wrocław, the congestion level was 31–36% lower than that in 2019. Our research results can be especially useful to city planners to help them locate bike routs more responsible to avoid heavily trafficked areas when possible. It is then necessary to build awareness that such a problem of health risk for cyclists exists, since most cyclists probably choose this type of transportation as a health and proactive stance not realizing that some routes are in fact “healthier” than the other.

Since there is an evident correlation between traffic intensity and contamination level of bike path dust with TRAP elements (especially almost entirely bioavailable and mobile Zn and Cd), it is therefore reasonable to avoid locating new cycling infrastructure in proximity to heavily trafficked main routes, especially near start/stop intersections. Simple solutions, such as avoiding cycling in traffic peak hours, are not applicable here, nor are they truly health protective measures. This is because this solid dust, when not physically removed from the bike path or roads, will most likely be resuspended and airborne, causing direct hazards to cyclists and the environment. Owing to dust contamination with heavy metals, which in approximately 90% results from nonexhaust sources such as abrasion of tires, clutches or brakes or even road surface infrastructure, it is therefore reasonable to physically remove dust by wet mechanical sweeping. Separating bicycle paths from the main routes in the city with green belts using specialized plants seems to also be a sensible approach, as it will improve the safety of cycling and, above all, will limit dust resuspension, thus reducing the potential health risk associated with exposure to dust for cyclists.
